# A symptomatic pelvic digit with surgical and pathological correlation

**DOI:** 10.1016/j.radcr.2025.02.104

**Published:** 2025-03-26

**Authors:** Romain Auger, Maxime Gouguet, Jean-Philippe Cottier, Valentin Lefevre

**Affiliations:** a Department of Radiology and Medical Imaging, CHRU Bretonneau, 2 Boulevard Tonnellé, 37000 Tours, France; b Department of Radiology and Medical Imaging, CHRU Trousseau, Avenue de la République, 37170 Chambray-lès-Tours, France

**Keywords:** Pelvic digit, Erectile dysfunction, Exostosis, Pudendal neuralgia

## Abstract

Pelvic digits (also known as pelvic fingers or pelvic ribs) are rare supernumerary benign bony lesions. Most of them are asymptomatic but, when symptomatic, they can pose a diagnostic challenge. We hereby present a case of a pelvic digit responsible for an organic erectile dysfunction and a disabling pain in the sitting position. Pelvic radiographs showed a 2-cm ossified cannulated structure emerging from the right ischiopubic ramus, extending down into the right perineal soft tissues.

MRI revealed a well-defined cortico-medullary digit with typical bone signal, developing near the hypertrophied root of the right corpus cavernosum and the insertion of the right adductor magnus muscle. The CT scan confirmed a pelvic digit with a pseudarthrotic single joint on the ischium. After a thorough 2-step surgical resection, pathologists confirmed the diagnosis. This particular radiologic challenge was to differentiate a pelvic digit from osteochondroma, avulsion-fracture sequelae, ligamentous calcification and myositis ossificans.

## Introduction

A pelvic digit should be considered in the differential diagnosis of various ossified structures in the pelvis (osteochondroma, avulsion-fracture sequelae, ligamentous calcification and myositis ossificans). Very few of them are symptomatic and are often incidentally discovered. Relevant medical history such as previous trauma, hematoma, previous medical imaging and patients’ complaints are an essential guide to the diagnosis. We hereby report, according to our knowledge, the first case of an erectile dysfunction caused by a pelvic digit.

## Case report

A 65-year-old farmer presented to the emergency department with right perineal pain, which worsened while sitting and during kneeling movements. The patient experienced significant discomfort while cycling or riding a motorcycle and he had decided to stop these activities many years ago. Medical history revealed a gnawing pain radiating to the right testicle and to the inferior inguinal area leading to an organic erectile dysfunction making it difficult to maintain a strong erection.

**Fig. 1:**
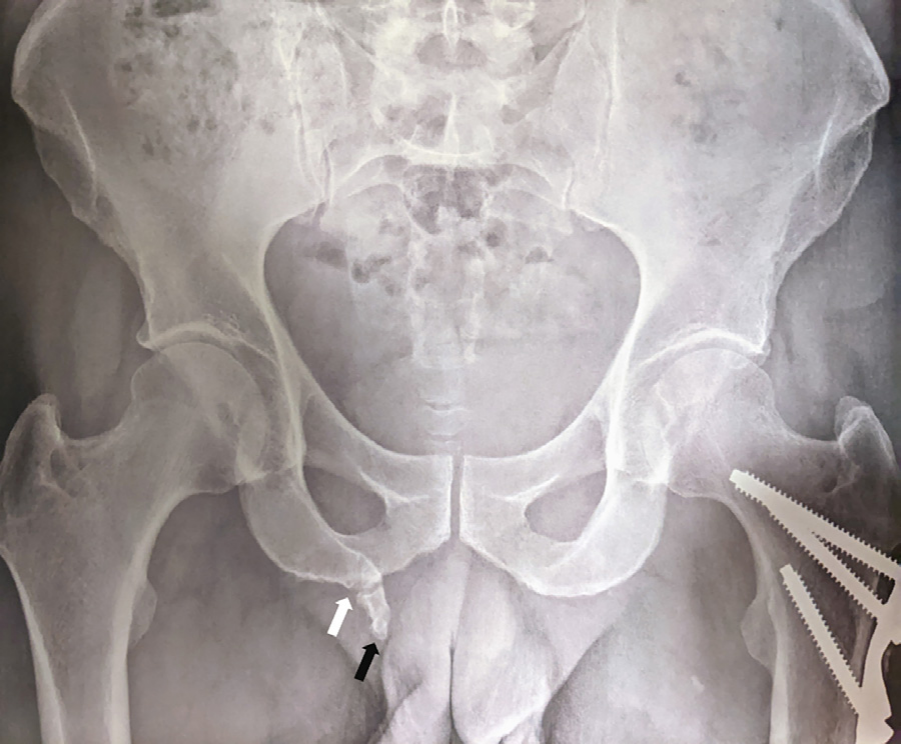
Pelvic radiography (frontal view). A mature pelvic digit, corticated (black arrow) extending from the right ischium. The proximal joint (white arrow) represents a pseudoarthrotic articulation with the ischiopubic ramus.

Given this clinical presentation, the initial hypothesis was pudendal neuralgia. Initially, a pelvic radiograph was prescribed, whose findings included a mature and cannulated 2-cm long bony protuberance attached to the right ischiopubic ramus, featuring an articulation at its origin, oriented toward the right perineal soft tissues (Fig. 1).

**Fig. 2:**
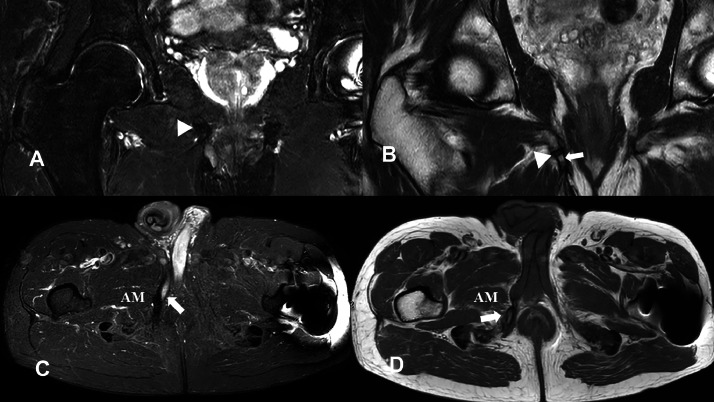
Pelvic MRI (1.5 Tesla). (A) and (B) Coronal T2 Dixon and T1-weighted images showing a pelvic digit with high T1 and low T2 signal intensity, consistent with cortico-medullary bone components (white arrow), and a proximal pseudoarthrotic joint with the ischiopubic branch (white arrowhead). (C) Axial STIR image demonstrating the extension of the digit towards the hypertrophied insertion of the right corpus cavernosum (white arrow). (D) Axial T1-weighted image showing direct contact with the insertion of the right adductor magnus (AM) muscle (white arrow).

As an exostotic lesion was suspected, an MRI was performed later. It showed an oblong cortico-spongious structure emerging from the antero-inferior margin of the right ischiopubic ramus, showing the same signal as the adjacent bone, i.e. increased T1 signal, decreased T2 and STIR signal (24 mm high, 8 mm antero-posterior diameter, 7 mm thick) (Fig. 2). It also confirmed the presence of a single joint with the right ischiopubic ramus and that one of its ends extended toward the bony insertion of the ipsilateral corpus cavernosum.

**Fig. 3:**
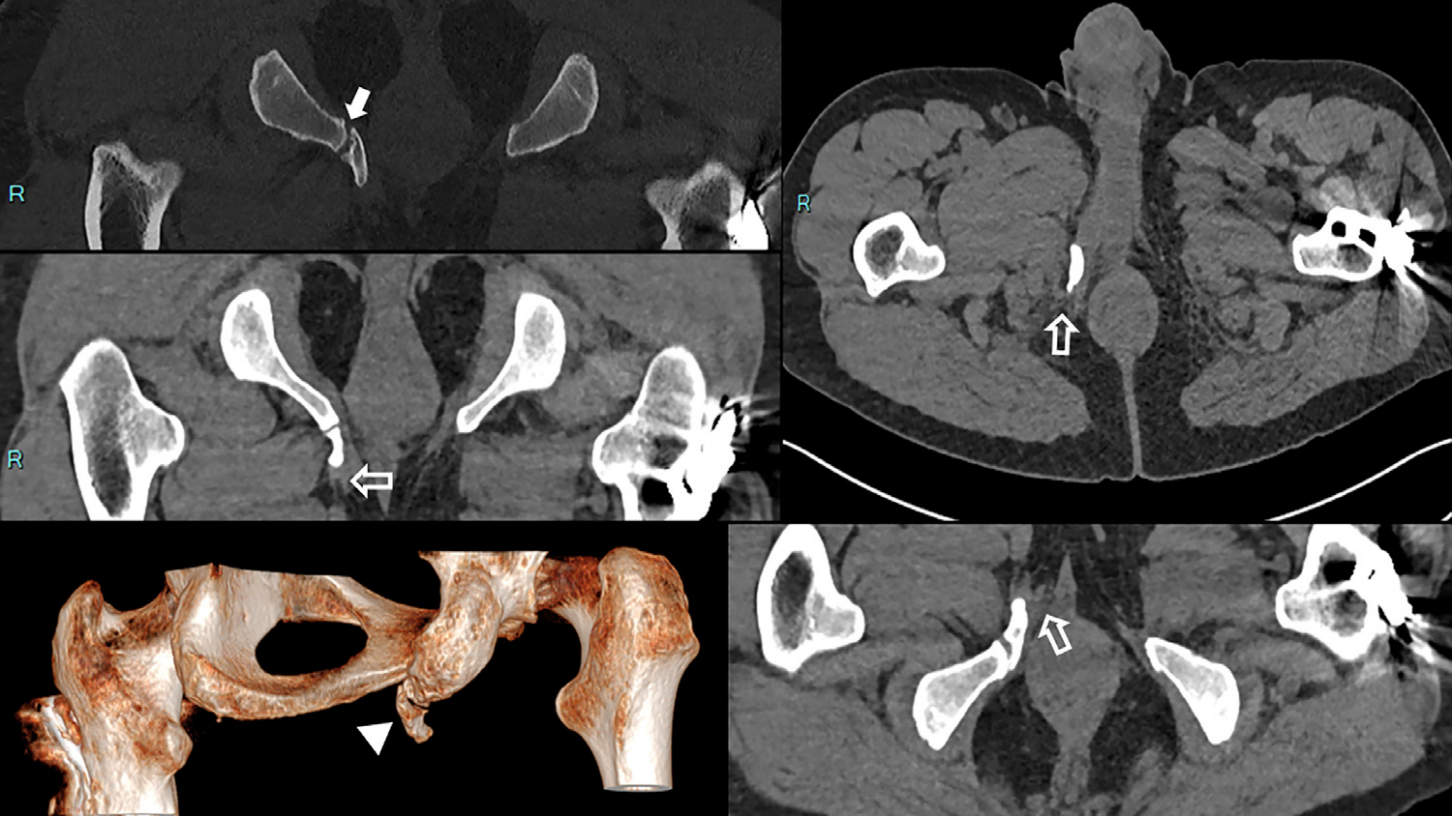
Pelvic CT scan. The pelvic digit extends anteriorly and demonstrates a pseudoarthrotic articulation with the right ischiopubic branch (white arrow). The 3D reconstruction (white arrowhead) highlights its anteroinferior orientation. Note the soft tissue infiltration around the distal end of the digit, particularly in the subcutaneous fat, likely due to chronic mechanical irritation in the sitting position (white underlined arrow).

**Fig. 4:**
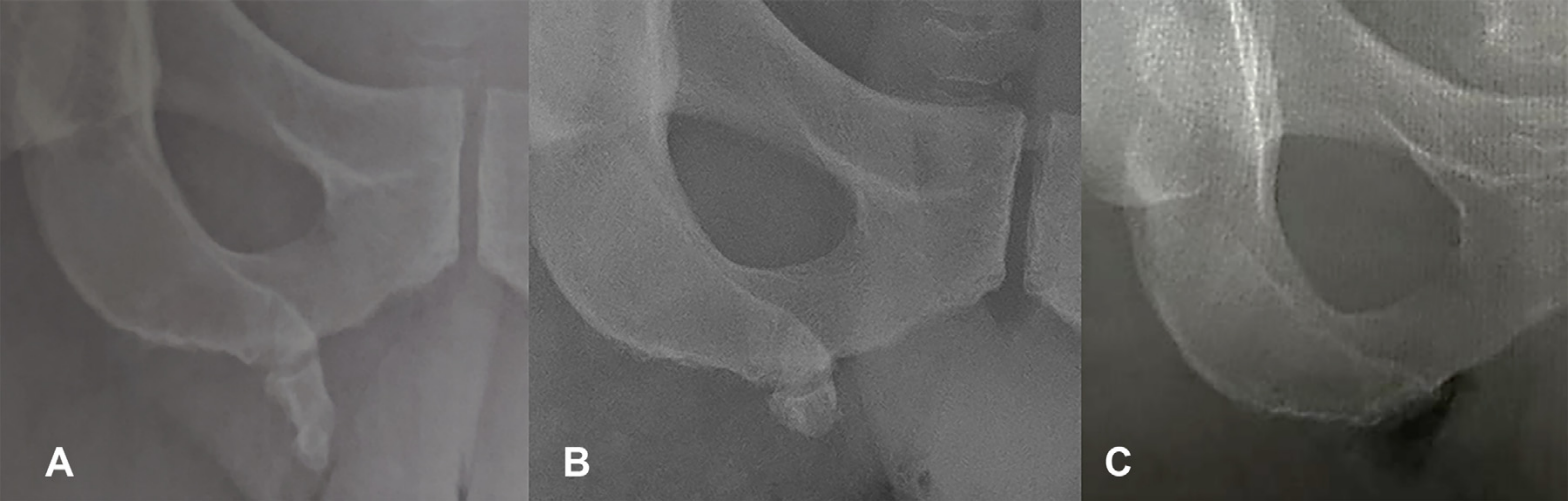
Pelvic radiographs (frontal view) before (A) and after (B) partial surgical resection of the pelvic digit. Complete resection was performed 9 months later (C).

The right corpus cavernosum was hypertrophied compared to the contralateral side, which we hypothesized to be caused by the mechanical constraint exerted by the ossified structure. Furthermore, it was deforming the pubic insertion of the right adductor magnus. There were no peripherical high T2 signals, ruling out a cartilage cap and the differential of osteochondroma. A pelvic CT confirmed the diagnosis of a pelvic digit, in contact with the right ischiopubic ramus, demonstrating a fully ossified bone with no cartilaginous cap (Fig. 3). The cortex of the ischium and the base of the pelvic digit formed a single pseudoarthrotic joint. Moreover, the CT confirmed a close relationship with the root of the right corpus cavernosum and the insertion of the right adductor magnus. It also revealed a focal infiltration of soft tissues surrounding the extremity of the digit, particularly in the hypodermic fat due to a chronic conflict in the sitting position. This specific symptom pattern is rarely observed. For this reason, we consulted a multidisciplinary team to assess the indication for surgical intervention, primarily to alleviate the patient's pain. The patient initially declined the surgical intervention. Two and a half years later, he consulted the surgeon again because the pain had increased, radiating to the right iliac fossa and the right flank. The patient requested surgical removal of the lesion because sitting had become unbearable. As there were no contraindications, a 2-step surgical procedure was performed, enabling the removal of the pelvic digit with a 9-month interval between the surgeries. The procedure involved the use of Liston bone-cutting forceps after incising the right adductor aponeurosis (Fig. 4).

The pathological analysis confirmed a mature ossification of the right ischium with no evidence of malignancy. Microscopic examination revealed well-differentiated lamellar bone covered by fibrocartilaginous tissue, along with a foreign body resorptive macrophagic granuloma, likely related to the previous biopsy. Altogether, the diagnosis was consistent with a pelvic digit.

## Discussion

Pelvic digits are rare congenital supernumerary bony structures developing in the pelvic soft tissues. Their origin remains unknown. The leading hypothesis suggests persistence of mesenchymal remnants during the first 6 weeks of embryonic development leading to unfused costal cartilage that develops independently as a rib-like structure on the pelvic bones [1]. Firstly, we must bear in mind that it constitutes a rare clinical occurrence, often incidentally discovered. The first case described in literature in 1974 was an asymptomatic pelvic rib in a young girl [2].

Second, it should be noted that only a few of them are symptomatic. A rare case of dyspareunia in a woman has been associated with a pelvic digit [3]. It is more frequently unilateral than bilateral, affecting mostly the ilium and ischium [4,5]. Although rare, this condition must be recognized to avoid unnecessary or invasive investigations.

Third, surgery is considered only in rare cases of symptomatic pelvic digits. If performed, the surgeon needs a thorough anatomical mapping in order to evaluate the benefit-risk balance of the surgery. In such cases, CT-scans and MRI are complementary to better evaluate the anatomical relationship with the surrounding tissues. Fourth, the diagnosis of a pelvic digit should be known and recognized, and we think that one must consider this diagnosis in case of a similar clinical and radiological pattern.

Differentiating a pelvic digit from the conditions listed below can be challenging.

Several differential diagnoses must be considered when identifying a pelvic digit, particularly osteochondroma, myositis ossificans, avulsion fracture sequelae, and ligamentous calcifications, each with distinct histopathological characteristics.

Osteochondromas, the primary differential diagnosis, are distinguished by a cartilage cap with endochondral ossification, absent in pelvic digits. They also exhibit direct continuity between the lesion's cortex and the parent bone, unlike the pseudoarthrotic joint observed in pelvic digits [6,7].

Myositis ossificans typically presents as a zonal pattern of ossification with a central fibroblastic core and peripheral mature bone. Unlike a pelvic digit, which exhibits well-differentiated lamellar bone with a cortico-medullary pattern, myositis ossificans lacks a true pseudoarticulation [8].

Avulsion fracture sequelae may contain reactive bone and fibrosis but often show irregular bone margins and healing trabeculae, contrasting with the organized bone structure of a pelvic digit.

Ligamentous calcifications appear as amorphous deposits of calcium hydroxyapatite within fibrous tissue rather than structured lamellar bone, and they do not exhibit a pseudoarticular connection to the adjacent bone.

Pelvic digits consist of mature, well-differentiated lamellar bone with fibrocartilaginous covering, lacking the reactive or neoplastic features seen in other conditions.

Finally, this case shows that the patient's clinical history (no prior trauma) and physical examination along with the imaging data reinforced by a multidisciplinary dialogue are crucial to provide the best possible care for the patient.

Given these findings, pelvic digits should be systematically considered in patients with similar clinical and radiological presentations.

## Conclusion

Pelvic digits are rare congenital anomalies that should be considered in the differential diagnosis of chronic perineal pain and erectile dysfunction. Multimodal imaging is crucial for characterization and differentiation from other ossified structures. In symptomatic cases, surgical resection can be beneficial, provided a thorough preoperative assessment is performed. A multidisciplinary approach ensures optimal patient management.

## Patient consent

The case report patient's authorizes in writing the healthcare professionals at CHRU Bretonneau and Trousseau of Tours to use the medical information and details of his condition for a case report intended to be published in a medical journal.

He understands that this publication may include specific details about his health condition, including medical images (X-rays, MRIs, etc.) and relevant clinical information to illustrate and explain the condition. He is aware that all necessary measures will be taken to protect his anonymity and his identity will not be disclosed in the publication.

He also acknowledges that, although all precautions are taken to protect his confidentiality, there remains a residual risk that individuals may identify his case based on the published information.

He voluntarily agrees to allow the use of his medical information for this publication, and he understands that his participation is entirely voluntary. He confirms that he had the opportunity to ask questions about this form and that all his questions have been satisfactorily answered.

By signing the form, he declares that he gives his informed consent for the publication of this case report.
